# Multidisciplinary rehabilitation intervention on mobility and hemodynamics of joint contracture animal model

**DOI:** 10.1186/s13018-023-03768-8

**Published:** 2023-04-13

**Authors:** Palida Maimaiti, Guhaer Aisilahong, Jiao Jiao Shu, Parhat Rozi, Nuerbiya Keqike, Tianyu Miao, Ningning Wang

**Affiliations:** 1 Nursing School of Xinjiang Medical University, Urumqi, 830011 China; 2 Xinjiang Agricultural University, Urumqi, 830052 China; 3 The First Affiliated Hospital of Xinjiang Medical University, Urumqi, China

**Keywords:** Joint contracture, ROM, Treadmill workouts, Multidisciplinary rehabilitation treatments, Hemodynamics

## Abstract

**Background:**

Joint contracture causes a decrease in range of motion (ROM), which severely affects activities of daily living of patients. We have investigated the effectiveness of a multidisciplinary rehabilitation on joint contracture by rat model.

**Methods:**

We used 60 Wistar rats in this study. The rats were divided into five groups as follows: group 1 was the normal control group; except the group 1, we created left hind limb knee joint contracture using Nagai method for other four groups. The joint contracture modeling group 2 was the model control group for monitoring the spontaneous recovery, and other three groups were given different rehabilitation treatments; for example, group 3 was treadmill running group; group 4 was medication group; group 5 was treadmill running plus medication group. The left hind limbs knee joint ROM and the femoral blood flow indicators (FBFI) including PS, ED, RI, and PI were measured right before and after the 4 weeks of rehabilitation.

**Result:**

After 4 weeks of rehabilitation treatments, the measured values of ROM and FBFI are compared with the corresponding values of group 2. Firstly, we did not see clear difference in the values of ROM and FBFI for group 2 before and after 4 weeks spontaneous recovery. The improvement of left lower limb ROM for group 4 and group 5 as compared to the group 2 was statistically significant (*p* < 0.05), whereas a less recovery for group 3 was observed. However as compared to the group 1, we did not observe full recovery in ROM of group 4 and group 5 after 4 weeks of rehabilitation. The PS and ED level for rehabilitation treatment groups was significantly higher than those modeling ones (Tables 2, 3,  Figs. 4, 5), while the RI and PI values show the contrary trends (Tables 4, 5, Figs. 6, 7).

**Conclusion:**

Our results indicate that multidisciplinary rehabilitation treatments had a curative effect on both contracture of joints and the abnormal femoral circulations.

## Introduction

The normal range of joint motion (ROM) is maintained by repeated daily movements. Immobilization is one of the major causes of joint contracture. Joint contracture is one of the main symptoms of disuse syndrome, which is caused by limitation of ROM [1]. Joint contracture causes a decrease in ROM [1–3]. Many studies report that after the formation of joint contracture, it is difficult to completely restore ROM to normal level even through a large number of conservative rehabilitation or surgical treatment are given [4–6]. Thus, stimulated controlled investigations of various treatment methods are necessary for progress in orthopedics [4, 7, 8]. Knee flexion contracture can be surgically treated by posterior soft tissue release such as hamstring lengthening, proximal gastrocnemius release, and posterior capsule release. Studies show that joint contracture mainly adopts comprehensive rehabilitation therapy such as exercise therapy, physical factor therapy, drug therapy, and orthosis. The above mentioned therapies have important advantages and clinical value in improving patients’ daily living ability and for reducing disability rate and alleviating symptoms [9–11].

Although the above research has a good clinical basis in the treatment of joint contracture, it still focuses on the improvement of ROM and ADL in patients, and there is little experimental work focused on the objective indicators of the pathogenesis of joint contracture. The pathogenesis of joint contracture and changes in peripheral blood flow are poorly understood, so understanding the pathophysiology of joint contracture and making full use of animal models is crucial. In this study, we have considered not only the limitation of ROM, but also the change of blood flow velocity when the joint contracted by immobilization. The aim of this study is to establish a joint contracture rat model and apply a multidisciplinary rehabilitation treatment method, including medication treatment using KERUTI ointment. This relaxant ointment is applied and used in the Hospital of Xinjiang traditional Uyghur Medicine, but more clinical trials and basic research are needed to provide theoretical support and scientific evidence for further application. In this study, we are not only studied the mechanism of the ROM of the knee, but also using Doppler ultrasonography to have studied blood flow velocity within the femoral artery, specifically, peak systolic velocity (PSV/AD, where AD refers to the abdominal aorta diameter), end-diastolic velocity (EDV/AD), vascular resistance index (RI), and pulsatility index (PI) [12–16].

## Material and methods

### Sample and surgical procedure

Sixty male 8-week-old Wistar rats with an initial body weight of 190–250 g were used. This study was performed according to the Regulations on Animal Experiments of the Xinjiang Medical University of China and was approved by the Animal Experiments Committee (Approval Number: SYXK (XIN) 2011-0003). The animals were housed in a temperature-controlled room at 22 °C on a 12-h light dark cycle. The rats had provided free access to standard rat food and water.

**Fig. 1:**
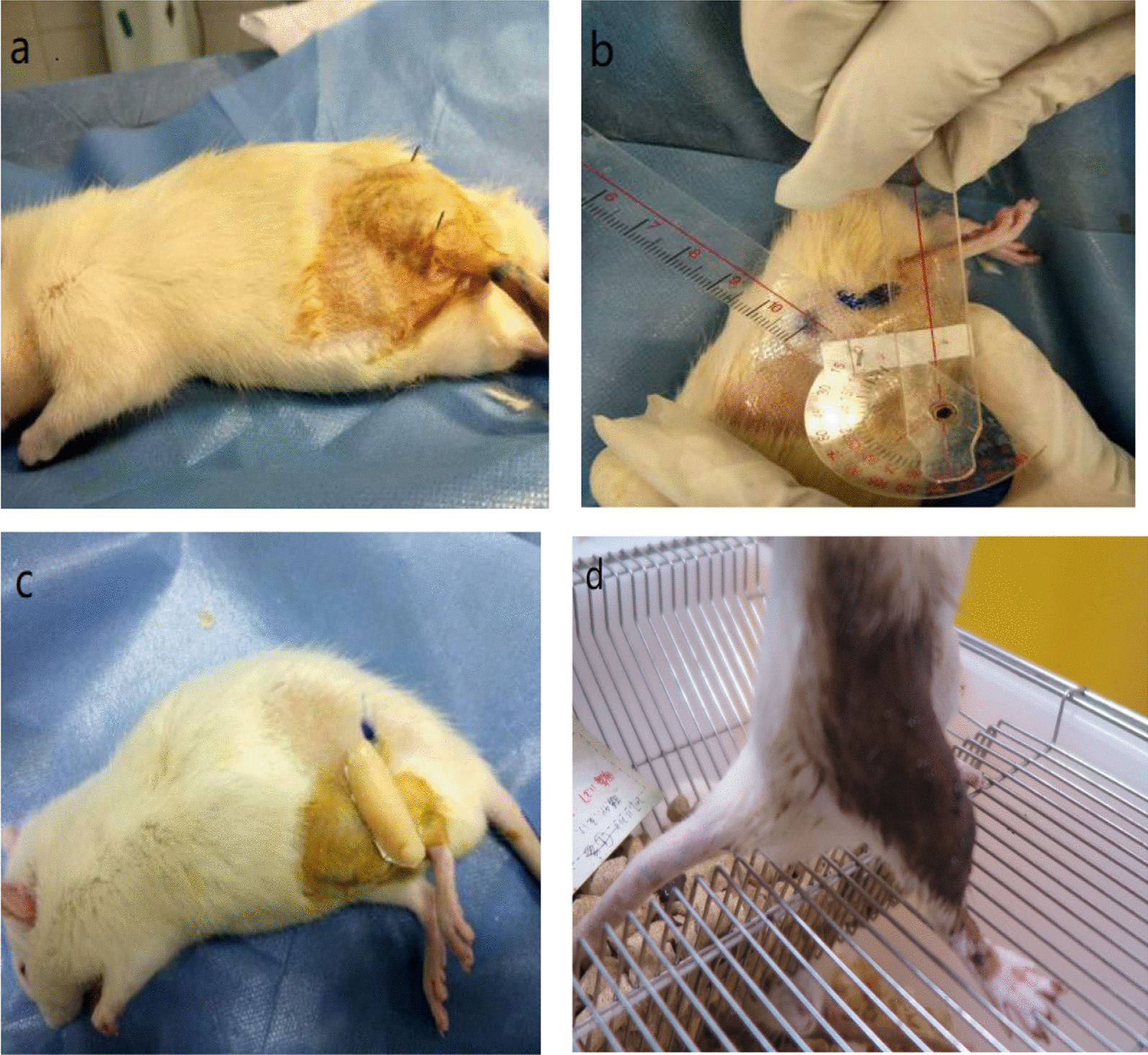
**a** position of Kirschner's needle **b** measuring ROM. **c** fixed. **d** put medicine on

Rats randomly allocated in groups of one control group (12rats) and four experimental groups (48rats). We have created joint contracture on the left hind limb knee of the rats in four experimental groups using Nagai method [17]. The left hind limb of each experimental animal was immobilized for 3 weeks [2, 17, 18] with an external fixator consisting of wire and resin. Under sodium Nembutal anesthesia and sterile conditions, Kirschner wires were screwed into the femur and the tibia and fixed with wire and resin to maintain knee flexion of approximately 140° ± 5° as shown in Fig. 1.

The normal control group (12rats) was named group 1. Four rats are dead with excessive anesthesia, and femur fractures appeared in four rats. These eight rats were excluded, and the other 40 rats were successfully organized in experimental groups. These model rats were randomly divided into 4 groups as model control group, treadmill running group, medication group using KERUTI ointment and massage, and intervention group, which were labeled as group 2, group 3, group 4, and group 5, respectively. Total 10 rats are in each group.

### Intervention method

#### Group 1 and Group 2

Group 1 was the model group. We did not produce knee joint contractures in the rats in this group and did not use any of the rehabilitation treatments. Except for group 1, we created left hind limb knee joint contracture using Nagai method for the other four groups. Group 2 was used as the model control group. No intervention was given to group 2 after fixator removal.

#### Group 3 (treadmill running group)

The rats are placed on a treadmill with four running tracks, one rat on one running track. The rats were jogging once a day on a treadmill at a speed of 1 km/h and a time set of 20 min. The speed of 1 km/h is a little bit faster than walking. After the exercise, the rats were returned to the cage.

#### Group 4 (medication group)

We have used the relaxation muscle ointment KERUTI as a topical medicine. KERUTI is Xinjiang characteristic relaxant ointment with all natural herbs, which is traditionally used for osteoarthritis therapy among the Chinese ethnic minorities in northwest China. We have carefully shaved off the hair of the rat's model side limbs to expose the skin around the knee joint. And then, we have applied KERUTI ointment to the pelvic, thigh, the inner and outer parts of the knee, and to the upper third of the lower leg (Fig. 1d). We applied a slight massage to the medicated area during the ointment process. After the medication process, the rats are returned to the squirrel cage, and the relaxant ointment stays on the limb for 4 h. We have performed the medication once a day and 7 days in a week.

#### Group 5 (rehabilitation intervention group)

The rats were placed on a treadmill, and the speed and time were the same as used for the group 3, and the KERUTI ointment was applied as the same routine for group 4 after the jogging was completed.

### ROM analysis

**Fig.2:**
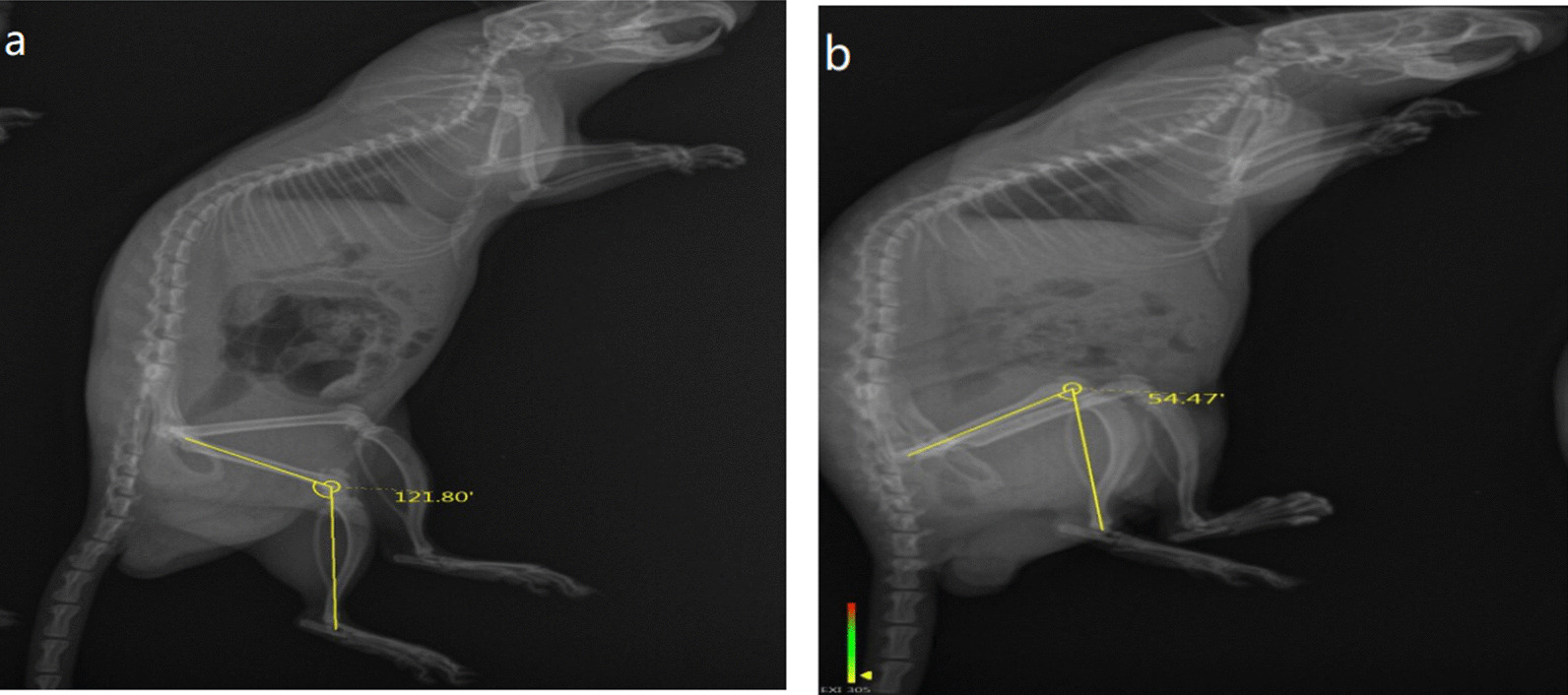
**a** Normal control group ROM(121.83°) **b** model control group(54.47°)

At the end of the immobilization period, the wire and resin were removed from the joint and ROM analysis was performed. Rats were anesthetized after fixator removal. X-ray tomography was taken for each rat to measure the left knee joint ROM. Thereafter, the ROM was calculated using the Image J software package (National Institutes of Health, USA) as shown in Fig. 2. The detailed measurement method of the ROM is as follows [6, 17]:

After anesthesia, the joint is placed in a horizontal position. The body of the rats is fixed manually to prevent sliding when a torque force applied to the ankle position of the limb. We have used a force gauge to ensure that the direction and tension applied are the same as in conducted measurements. The spring head of force gauges was attached to the distal part of the ankle, and then, the strings were pulled with a tension of 0.45 N.

### Detection of femoral artery blood flow index

**Fig.3:**
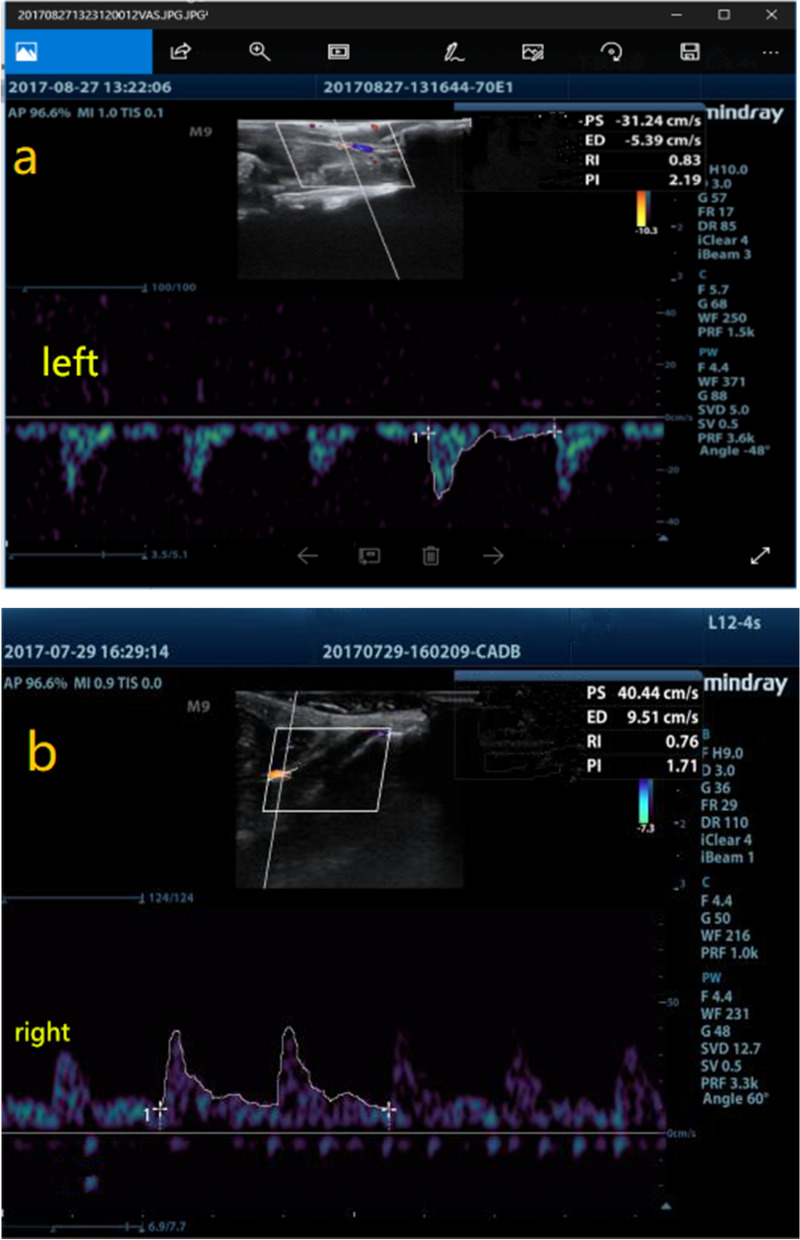
**a** Blood flow waveform of the affected femoral artery. **b** Contralateral femoral artery blood flow waveform

We have used the M9Vet portable color Doppler ultrasound system. Before measurements, the limb stretched, and we have shaved the hind limb hair of rats. The probe area on the knee joint is from 3 to 4 cm. Four indicators of femoral blood flow were measured on the blood flow waveform in the Doppler image, namely PS, ED, RI, and PI, as shown in Fig. 3.

### Statistical analysis

**Fig. 4:**
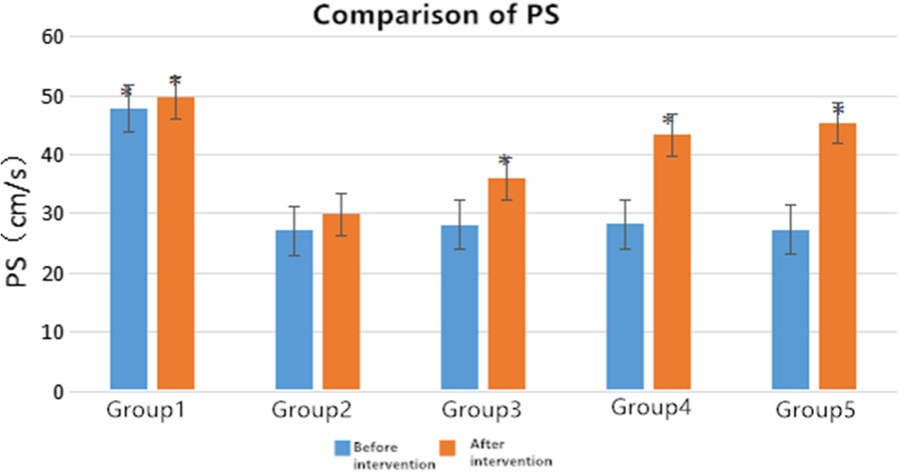
Changes of PS in each group before and after intervention. *Significant contribution to compared with the group 2, *p* < 0.05

**Fig. 5:**
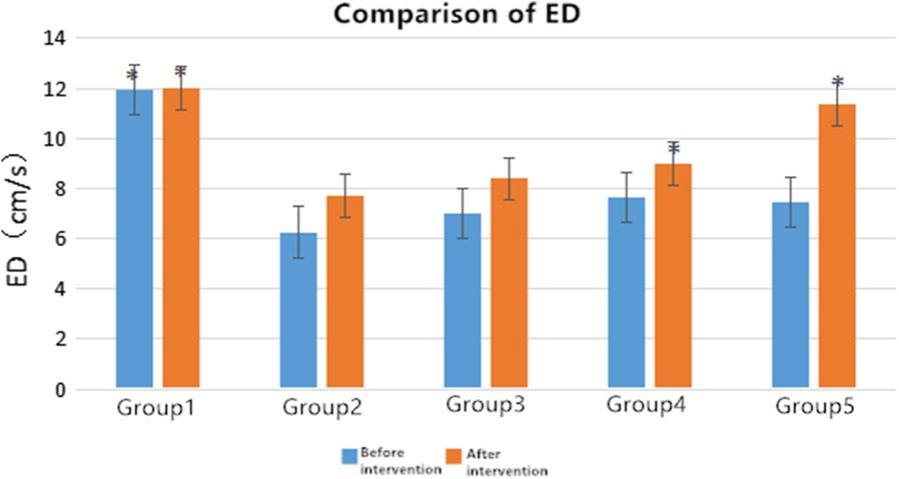
Changes of ED in each group before and after intervention. *Significant contribution to compared with the group 2, *p* < 0.05

**Fig. 6:**
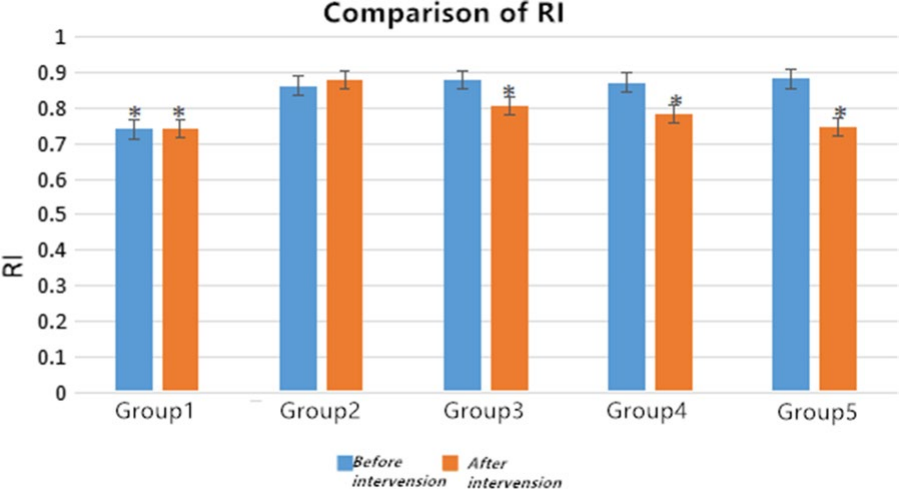
Changes of RI in each group before and after intervention. *Significant contribution to compared with the group 2, *p* < 0.05

**Fig. 7:**
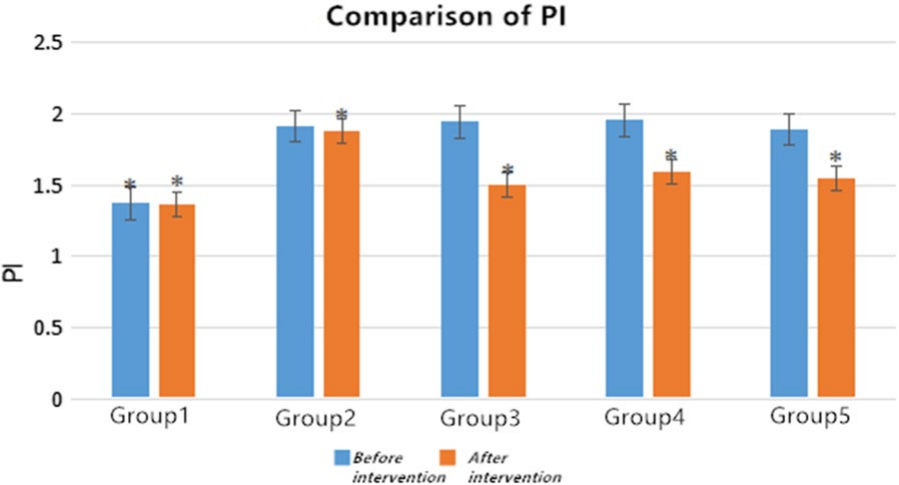
Changes of PI in each group before and after intervention. *Significant contribution to compared with the group 2,* p* < 0.05

At least five samples for each experimental condition were analyzed. All values are presented as mean ± standard deviation (SD). Analyses were performed with SPSS 22.0 J for Windows. We have carried out multicomparison analysis using one-way analysis of covariance (ANCOVA) for ROM. And PS, ED, RI, PI were compared among all groups using the one-way analysis of variance (ANOVA) test. It was utilized to analyze the data followed by the *t*-test. An alpha less than 0.05 was chosen as the significance level for these statistical analyses Figs. 4, 5, 6, 7.

## Results

### Changes in ROM before and after intervention

The results of paired *t*-test showed that there is significantly reduce in ROM of four experimental groups compared with the group 1 before the intervention. The difference of the ROM between group 1 and other four groups was statistically significant (*p* < 0.05), indicating clear knee contracture happened in four experimental group. We did not see clear difference in the values of ROM for group 2 before and after 4 weeks spontaneous recovery. After 4 weeks of intervention, we also did not see statistically difference (*P* = 0.082 > 0.05) between group 2 and group 3 indicating jogging on treadmill does not help to improve the ROM. The improvement of left lower limb ROM for group 4 and group 5 as compared to the group 2 and group 3 were statistically significant (*p* < 0.05), whereas a less recovery for group 3 was observed. However, as compared to the group 1, we did not observe full recovery in ROM of group 4 and group 5 after 4 weeks of rehabilitation. We did not see statistically difference (*p* = 0.328 > 0.05) between group 4 and group 5 (Table 1).

**Table 1:** Comparison of ROM intervention in different groups of Rats(‾X ± S)

Groups	Left lower limb ROM (Degree)	
	Before intervention	After intervention
Group 1	109.7 ± 5.74	111.72 ± 3.85
Group 2	74.74 ± 9.23*	72.20 ± 12.73
Group 3	72.32 ± 7.79*	78.13 ± 7.07
Group 4	71.75 ± 8.33*	91.38 ± 11.20#
Group 5	74.10 ± 6.77*	103.54 ± 4.36#

^#^Significant improvement compared with the group 2 and group 3, *p* < 0.05. *A clear knee contracture is achieved as compared to group 1, *p* < 0.05

### Changes of blood flow index of rats in each group before and after intervention

The *t*-test indicates that after intervention the values of PS, ED, RI, and PI in each group were higher than those values of before intervention (*p* < 0.05). Especially, after 4 weeks of intervention, PS and ED levels were significantly higher than the values of before intervention (Tables 2 and 3), comparable to the values of group 1 indicating clear medication effect on these two-blood flow velocity, whereas the RI and PI values show on a contrary trend (Tables 4 and 5).

**Table 2:** Comparison of peak systolic blood flow velocity PS in each group

Groups	PS (cm/s)		*t value*	*p value*
	Before intervention	After intervention		
Group 1	47.812 ± 3.473	49.731 ± 3.296	− 1.327	0.226
Group 2	27.105 ± 3.956	29.874 ± 4.065	− 2.341	0.052
Group 3	28.165 ± 5.011	35.950 ± 5.458	− 3.187	0.015
Group 4	28.233 ± 3.974	43.423 ± 2.896	− 10.184	0.001
Group 5	27.344 ± 4.703	45.406 ± 8.345	− 4.658	0.002

**Table 3:** Comparison of end-diastolic velocity ED in all groups

Groups	ED (cm/s)		*t value*	*p value*
	Before intervention	After intervention		
Group 1	11.959 ± 1.504	12.001 ± 1.899	− 0.065	0.95
Group 2	6.260 ± 2.191	7.695 ± 0.336	− 1.888	0.101
Group 3	6.981 ± 1.926	8.391 ± 2.285	− 1.233	0.257
Group 4	7.631 ± 0.352	8.977 ± 0.997	− 4.144	0.004
Group 5	7.446 ± 0.608	11.372 ± 3.402	− 3.275	0.014

**Table 4:** comparison of resistance index RI among groups

Groups	RI		*t value*	*p value*
	Before intervention	After intervention		
Group 1	0.741 ± 0.0349	0.741 ± 0.058	0	1
Group 2	0.863 ± 0.066	0.880 ± 0.043	− 0.552	0.598
Group 3	0.880 ± 0.034	0.805 ± 0.047	3.08	0.018
Group 4	0.873 ± 0.045	0.784 ± 0.049	3.309	0.013
Group 5	0.884 ± 0.044	0.749 ± 0.053	6.339	0.0001

**Table 5:** comparison of resistance index PI among groups

Groups	PI		*t* value	*p* value
	Before intervention	After intervention		
Group 1	1.371 ± 0.0136	1.364 ± 0.017	1.528	0.17
Group 2	1.914 ± 0.129	1.883 ± 0.103	0.498	0.634
Group 3	1.944 ± 0.182	1.496 ± 0.236	3.757	0.007
Group 4	1.956 ± 0.163	1.595 ± 0.232	3.292	0.013
Group 5	1.889 ± 0.090	1.543 ± 0.290	4.035	0.005

## Discussion

The mechanics of blood flowing in the cardiovascular system is called hemodynamics. Blood flow parameters reflect the stiffness of the aorta and the vascular resistance of the arterial tree distal to the point of measurement. Aortic stiffness is associated with microvascular damage in target organ tissues [13–16]. Thus, microvascular damage in target organ tissues has a corresponding relationship with blood circulation disorders. As far as the nature of the disease is concerned, it is mainly local or mainly systemic. During the course of the disease, local and overall reactions interact, and the local blood circulation disorders affect the systemic blood circulation, and blood circulation disorders are related to the cause of hemodynamic changes. Another purpose of this study is to find out whether there is a femoral artery circulatory disorder in the knee joint contracture in rats.

In this study, treadmill exercise was first used as a simple intervention group (group 3), and then KERUTI was used as another simple drug intervention group (group 4). KERUTI is a traditional Chinese medicine, made into an ointment according to the Uyghur medical compatibility method (Uyghur medicine is part of traditional Chinese medical science). This ointment has the effect of improving muscle tension and spasm around the joints, enhancing the flexibility of the joint ligaments, reducing the joint rubbing and improving the range of joint mobility. The drug is applied and used in the Hospital of Xinjiang traditional Uyghur Medicine, but more clinical trials and basic research are needed to provide theoretical support and scientific evidence. The treadmill running plus medication group was used as a rehabilitation intervention (group 5) to explore the efficacy among the three groups. In this study, we have found that when ROM is severely limited, we cannot get the ideal effect using simple exercise alone, but KERUTI has certain curative effect, and each rat has different degree of motion in a short 4-week period. Medication and rehabilitation intervention had a better effect on improving ROM of joint contracture limbs (see Table 1).

It was found that the decrease in ROM can be correlated with the decrease in PS value and ED value of the affected limb artery and with the increase in RI and PI. For the experimental four groups, the blood flow velocities are slowed down indicating all experimental model groups were obviously related to circulatory system pathologies. The results of treatment also showed that different intervention methods improved both ROM and hemodynamics. The PS and ED in the femoral artery of the left lower extremity increased after an intervention, especially in the medical group (group 4) and the rehabilitation intervention group (group 5) (Table 2 and 3). It can be seen from Tables 4 and 5 that all three interventions (group3, group4, group5) result a decrease in RI and PI index with statistical significance. The information obtained from Fig. 4 is that the model control group (group 2) has the lowest PS, while the group 4 and group 5 have a values higher than 40 (cm/s), which is close to the PS range of normal control group (group 1). It can be seen from Fig. 5 that after the intervention, the ED of all intervention groups are gradually increased. The rehabilitation intervention has a more obvious effect on the affected side.

RI assesses the integrity of pulsatile blood flow and reflects the resistance to blood flow or the functional integrity of the microvasculature distal to the measurement site. A typical RI for healthy young individuals ranges between 0.59 and 0.70. Higher values are consistent with hemodynamic stress or pathology [15]. This study constitutes novel findings regarding central hemodynamic responses to three adopted intervention methods, and it can provide that after intervention, the RI and PI of all intervention groups were reduced; the rehabilitation treatments in the group 5 have a more obvious effect on the affected side. Therefore, rehabilitation intervention is most effective in the treatment of left knee joint contracture in rats.

## Conclusion

From the results, it can be inferred that when the joint contraction occurs in the limb, it is not only necessary to strengthen the training of the limb, but also the need for a suited antispasmodic drug when the combination of befitting medicine and exercise is used as a rehabilitation measure, which can both beneficial to improve the range of motion of the limb joint as well as the blood circulation. Therefore, when joint contracture occurs in a certain joint part of an individual, it is extremely important to examine the hemodynamic changes as an observation index.

## Limitations

The analytic power of the current study was limited by the low number of experimental animals. Future studies with large animal groups would further deepen our understanding of the effects of comprehensive rehabilitation on hemodynamics of joint contracture in animal model, and there is less standard to assessment the animal model of joint contracture. We still have not cleared the mechanism of comprehensive rehabilitation effect on the joint contracture.
